# Can poor nutrition and diet influence temporomandibular disorder? A systematic review

**DOI:** 10.1186/s12903-026-08975-3

**Published:** 2026-06-23

**Authors:** Flávio Fidêncio de Lima, Artin Razavian, Mai Hosokawa, Tatiana Ferreira Foscaldo, Melisa Koldzo, Rodrigo Lorenzi Poluha, Maria Christidis, Nikolaos Christidis, Giancarlo De la Torre Canales

**Affiliations:** 1 Department of Dental Sciences, University Paulista, São Paulo, Brazil; 2 Division of Oral Diagnostics and Rehabilitation, Department of Dental Medicine, Karolinska Institutet, 14104 Huddinge, Sweden; 3 Department of Prosthodontics and Oral Rehabilitation, Nihon University School of Dentistry at Matsudo, Tokyo, Japan; 4 Postgraduate Program in Temporomandibular Disorders and Orofacial Pain, Kikuchi Institute, Pará, Brazil; 5 Department of Dentistry, State University of Maringa, Parana, Brazil; 6 The Swedish Red Cross University, The Institute of Health Sciences, Huddinge, SE-141 21 Sweden; 7 Egas Moniz Center for Interdisciplinary Research (CiiEM); Egas Moniz School of Health & Science, Caparica, Almada Portugal

**Keywords:** Chronic pain, Diet, Nutrition, Pain

## Abstract

**Background:**

The relationship between diet, nutrition, and painful temporomandibular disorders (TMD) is not well established, despite increasing interest in lifestyle factors in chronic pain. This systematic review aimed to synthesize available evidence on how dietary patterns and nutritional status may be associated with painful TMD.

**Methods:**

An electronic search was conducted in the databases MEDLINE, CINAHL, EMBASE, the Cochrane Central Registry of Controlled Trials (CENTRAL) and Web of Science for clinical and observational trials from the beginning of each database to November 2025. Risk of bias was performed using the Study Quality Assessment Tools from the National Heart, Lung, and Blood Institute (NHLBI).

**Results:**

Out of 7,760 records and after the risk of bias assessment, only seven studies judged as having a low risk of bias were included. Overall, the included studies indicate that the association between dietary factors and TMD remains unclear. Prospective cohort studies did not demonstrate that prenatal or adolescent diet quality independently predicted TMD. Cross-sectional studies suggested that specific dietary patterns may be related to TMD-related symptoms. Women with myogenous TMD showed lower intake of several nutrients compared with controls. Experimental evidence indicated that monosodium glutamate ingestion increased pain intensity in myofascial TMD, while the only randomized trial found no significant pain differences between vitamin D supplementation and diclofenac.

**Conclusions:**

Current evidence suggests that some dietary components and nutritional factors may be associated with TMD-related symptoms or pain responses; however, the findings are limited, heterogeneous, and insufficient to support firm causal or therapeutic conclusions.

**Supplementary Information:**

The online version contains supplementary material available at 10.1186/s12903-026-08975-3.

## Introduction

The World Health Organization (WHO) emphasizes nutrition as a key component of a healthy lifestyle and recognizes dietary patterns as major modifiable determinants of chronic disease [1, 2]. Evidence indicates that changes in diet can exert both beneficial and detrimental effects on health throughout life [3, 4]. Poor nutritional status and unhealthy dietary behaviors are therefore considered one of the most important lifestyle factors associated with chronic diseases [5] and have also been suggested to contribute to the onset and persistence of painful chronic musculoskeletal disorders [6, 7]. Lifestyle factors including obesity, excessive caloric intake, and diets rich in sugar, fat, sodium, and caffeine are frequently observed in individuals with chronic pain [8]. Moreover, certain dietary patterns and nutritional components have been proposed to influence systemic inflammation and pain perception, suggesting that nutrition may play a role in the modulation of chronic pain conditions.

Temporomandibular disorders (TMD) represent the most common chronic pain condition in the orofacial region and have the potential to evolve into persistent disorders [9]. The etiopathogenesis of TMD is multifactorial and involves complex interactions among biological, psychosocial and genetic factors [10]. Central sensitization represents a key neurophysiological mechanism frequently observed in individuals with TMD [58]. Emerging evidence suggests that neuro-immune mechanisms may link dietary intake with processes involved in pain modulation, including central sensitization inflammation and oxidative stress [11, 12]. Studies evaluating dietary patterns have shown that certain nutritional profiles may exhibit pro- or anti-inflammatory properties such as Mediterranean diet, (rich in omega-3 fatty acids and fibers), which have been pointed out as more favorable for reducing pain symptoms [7, 13–16]. In this context, nutrition has been proposed as a potential modulator of biological processes relevant to chronic pain conditions including TMDs [17–19].

In addition to potential biological mechanisms, painful TMDs may also influence dietary habits and nutritional intake [18]. The face and oral cavity play a fundamental role in communication, sensory perception, and food intake; therefore, disturbances in oral function may affect nutritional status. Painful TMDs can cause functional limitations during mastication and swallowing, altering the eating experience and influencing food choices. Patients with TMD often adopt adaptive behaviors to minimize pain, such as modifying food preparation or restricting the intake of foods that are difficult to chew, strategies that are also commonly recommended episodes of acute TMD pain [18, 20]. These changes may result in reduced intake of macro- and micronutrients, compromising overall health and nutritional status. Evidence also suggests that the frequent consumption of hard foods is positively associated with symptoms such as clicking and pain in the temporomandibular joint [21]. Moreover, ingestion of specific additives, such as monosodium glutamate (MSG), has been shown to increase glutamate concentration in the masseter muscle and exacerbate pain intensity in patients with myofascial TMD [22]. Painful TMD patients have also been reported to present lower scores in the Health Eating Index [23]. On the other hand, some dietary modifications including gluten-free diets or targeted micronutrient supplementation have been associated with reductions in myofascial TMD pain [24, 25].

Despite growing interest in the potential relationship between diet, nutrition, and chronic pain, the interaction between nutritional factors and TMDs remains poorly understood. Limited research has investigated how dietary intake may influence the development or maintenance of TMD, as well as how TMD symptoms may alter dietary behaviors and nutritional status. A better understanding of this relationship could provide new perspectives for both research and clinical practice, particularly considering the modifiable potential role of dietary assessment and nutritional guidance in the management of chronic pain conditions. Therefore, the aim of this systematic review is to synthesize the available evidence regarding the relationship between diet, nutrition, and TMD. Specifically, this review seeks to evaluate how dietary patterns, nutritional status, and specific dietary components may be associated with the presence, severity, or progression of TMD.

## Materials and methods

### Protocol and research question

This systematic review was registered a priori in Prospero (the International Prospective Register of Systematic Reviews, with registration number CRD420223469394) and was reported in accordance with the Preferred Reporting Items for Systematic Reviews and Meta-Analyses (PRISMA 2020) statement (Supplemental file 1). The research question was formed using the PECO framework [26], being an acronym for: P = Patients, E = Exposure, C = Control, O = Outcome, T = Time. In adults and adolescents (P) are diet and nutritional status associated (O) with painful temporomandibular disorders (E) compared to healthy individuals (C)?

- (P) Patients: Adults and adolescents that experience painful TMD.
- (E) Exposure: Primary research examining diet patterns and nutritional status in painful TMD.
- (C) Comparator: Healthy individuals not suffering from painful TMD.
- (O) Outcome: Clinical outcomes of painful TMDs (signs and symptoms).

The inclusion criteria that were applied were a)clinical trials (prospective, retrospective, observational, cross-sectional and randomized controlled trials (RCT)); b) adolescents and adult individuals presenting TMD. Exclusion criteria were a) studies that cannot be found in other languages other than English, Spanish, Portuguese or Scandinavian languages (Danish, Norwegian and Swedish); b) publications using duplicate data; c) editorials, letters, legal cases, interviews, case-series, case reports and reviews.

### Search strategy and study selection

The search strategy was developed and performed in MEDLINE (Ovid) in collaboration with the librarians AEM and LL at the Karolinska Institutet University Library. The search strategies were then peer-reviewed by the authors GDC and NC before the final searches. Authors identified each search concept using the Medical Subject Headings (MeSH-terms) and free text terms. Finally, the librarians translated the search into the other databases using the Polyglot Search Translator [27]. The final electronic search was performed in MEDLINE, EMBASE, CINAHL, CINAHL (esbsco) the Cochrane Central Registry of Controlled Trials (CENTRAL), and Web of Science databases from the inception of each database to April 2024. An update of the electronic search was performed in November 2025. De-duplication was performed using the method described by Bramer et al., (2016) [28]. Lastly, one final step was added to compare digital object identifiers (DOI) as well as a search in the reference-lists of the included studies and in several systematic reviews found in the search was performed. However, this did not result in any more full texts to include. The complete search strategy (for all databases) is available in Supplemental file 2.

To avoid any risk of biasness in the process of screening the studies, the web-based tool Covidence was used [29]. This was done independently and in a blind mode by two of the authors (AR and TF). In cases when there was disagreement regarding eligibility this was resolved by discussion with the author (GDC) who served as a judge making the final decision. When all disagreements were resolved the authors (AR and TF) attempted to retrieve the full texts of the included and potentially eligible studies. The studies that were retrieved were then reviewed in full text by the same authors to determine whether they aligned with the inclusion criteria or not. As before, any disagreement was resolved through a discussion with the author (GDC).

### Data extraction

A data extraction form was developed and tested independently on three randomly selected studies by two of the authors (FFL and MH) to ensure consistency in extraction. The extracted data included information on the characteristics of the included studies and study participants, such as the authors, diagnosis/criteria, mean age of patients, male–female ratio, nutritional/dietary interventions, timepoints for the follow-ups, and outcome measures. Any conflict in the data extracting process was resolved by the author (NC), who had the role of a judge.

### Analysis of risk of bias

Risk of bias was performed using the Study Quality Assessment Tools from the National Heart, Lung, and Blood Institute (NHLBI). https://www.nhlbi.nih.gov/health-topics/study-quality-assessment-tools) [30]. These tailored quality assessment tools serve as instruments to identify potential methodological or implementation-related limitations, e.g., aspects central to a study’s internal validity. Since the included studies were of different study designs, the following four instruments were used: a) Quality Assessment of Controlled Intervention Studies [24, 31–34]; b) Quality Assessment Tool for Before-After (Pre-Post) Studies With No Control Group [35–37] c) Quality Assessment Tool for Observational Cohort and Cross-Sectional Studies [23, 38–43]; and d) Quality Assessment of Case–Control Studies [7, 44]. Thus, the assessment was done on the level of parameters where minor or major flaws could be identified. Two authors (RLP and MK) evaluated the risk of bias for each study in a blind and independent manner. In cases where conflict arose it was resolved by discussion with the author (MC), who served as a judge. The individual quality assessment of each is presented in Supplemental file 3. Following quality assessment, we elected to restrict the synthesis to studies rated as “Good” during the review process, to provide a synthesis based on the highest-quality available evidence.

## Results

### Literature search outcome

**Fig. 1:**
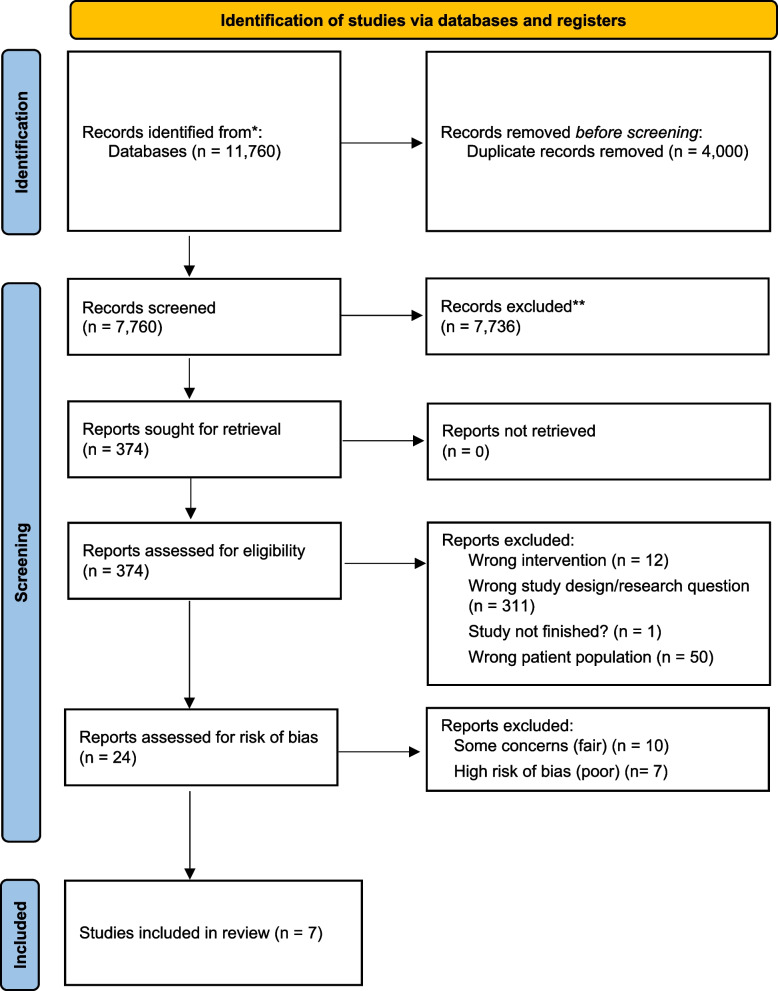
A Prisma flow diagram showing the retrieved and included records

The literature search initially identified 11,760 studies across all databases. After removal of duplicates, 7,760 records remained and were screened based on titles and abstracts. Of these, 374 articles were assessed in full text for eligibility. Following the risk of bias assessment, only studies judged as having a low risk of bias (Good) were retained. Consequently, of the 24 studies assessed, seven studies fulfilled the inclusion criteria and were included in the final review [21, 22, 45–49] A flow diagram illustrating the study selection process is presented in Fig. 1.

### Study characteristics, individual data, and risk of bias

Of the 24 studies assessed for risk of bias, seven were judged to have a low risk of bias and were included in the final review (Table 1). The characteristics of the included studies are summarized in Table 2. The included studies comprised a mixture of observational and experimental designs, including cross-sectional studies, prospective cohort studies, and one randomized controlled trial.

**Table 1:** Presentation of the summary of risk of bias of the 24 studies assessed using the Study Quality Assessment Tools of the National Heart, Lung, and Blood Institute (*for full checklist please see supplemental *Table 2). Only the 7 studies with low risk of bias (e.g. Good) were included in the review

Author	Risk of bias judgement
Aktas et al., 2023 [39]	Fair
Akhter et al., 2004 [21]	Good
Brennan et al., 2012 [45]	Good
Buosi et al., 2021 [24]	Poor
Catunda et al., 2016 [37]	Fair
Chlebowski et al., 2011 [38]	Fair
Di Giacomo et al., 2024 [34]	Fair
Exposto et al., 2026 [46]	Good
Gupta et al., 2022 [31]	Fair
Hedaya et al., 2017 [35]	Poor
Izham Akmal et al., 2020 [44]	Poor
Kahraman and Tunç, 2025 [47]	Good
Kesidou et al., 2024 [43]	Poor
Mansoori et al., 2025 [48]	Good
Marques et al., 2025 [23]	Fair
Nogueira et al., 2025 [24]	Good
Olson et al., 2000 [36]	Poor
Raphael et al., 2002 [7]	Fair
Riley et al., 2007 [41]	Fair
Sanders et al., 2025 [42]	Fair
Seltzer et al., 1982 [32]	Poor
Shimada et al., 2016 [22]	Good
Sigurdsson et al., 2024 [40]	Poor
Stockstill et al., 1989 [33]	Fair

**Table 2:** The extracted study characteristics of the seven included randomized controlled trials

Authors	Study design	Subgroup diagnosis	Criteria used	Age of patients	Male/Female	Intervention and Diet assessments	Results	Follow-up time
Akhter et al., 2004 [21]	Cross sectional	TMD (clicking sounds, jaw deviation, muscle and TMJ tenderness and TMJ pain)	Questionnaires Clinical examinations	12 to 17 years (mean 14.5 ± 1.7)	**Total sample:** 1200 **Women:** 602 (50.2%) **Men:** 598 (49.8%)	Questionnaire on food consumption for dietary intake TMD symptoms and hard food intake relationship: hard foods checklist divided into four categories: hard meats, hard vegetables/fruits, hard candies and nuts/dried grains	No significant relationship was found between TMD symptoms and dietary habits. There was no significant difference in clinical signs in relation to dietary habits A negative correlation (OR = 0.46) was found between intake of protrein hard food and restricted mouth opening Frequent consumption of hard vegetables and fruits (> 3 times/week) correlated with joint clicks. The high total consumption of all hard items (> 12 times/week) was significantly associated with increased prevalence TMJ pain	No
Brennnan et al., 2012 [45]	Cross sectional	Orofacial pain (jaw, temple, front of the ear and ear)	5 points Likert scale: sometimes/often/very often and never/hardly ever	**60 to 64:** 60.8% **65 to 71:** 39.2%	**Women:** 52.3% **Men:** 47.7%	Compliance with dietary guidelines by 16 items classified as "recommended" and “regular". Following the Australian National Health and Medical Research Concil	Worse overall health was significantly associated with: *low dietary compliance* (\beta = −0.031); *orofacial pain* (\beta = −0.042)	No
Exposto et al., 2026 [46]	PLCS	Painful and no TMD	Long version of TMD Screener	18 and 23 years	**Total sample:** 10.382 **Women:** 6674 (64.3%) **Men:** 3708 (35.7%)	FFQ: Of 360 items applied to mothers in the 25thª week of pregnancy, converted into a HEI based on Danish guidelines	There was no significant association between prenatal diet and painful TMD	1
Kahraman and Tunç, 2025 [47]	RCT	Myofascial pain	DC/TMD	Mean age: 24.8	**Total sample:** 40 **Women:** 37 (92.5%) **Men:** 3 (7.5%)	**G1:** splint/diclofenac sodium 75 mg 2 × 1 for 2 weeks **G2: splint/**10,000 IU of Vitamin D 1 × 1, for 8 weeks Pain with VAS and mouth opening measurements 25(OH)D levels	Subjective pain: no significant differences were found between groups at any follow-up Higher values were found for G2 in mandibular assisted opening at 7 days and 1 month assessment	7 days, 1 and 3 months
Mansoori et al., 2024 [48]	PLCS	Painful TMD No TMD	TMD: Long form of TMD screener	Specifically, we utilized the cohort of singletons who completed the 14-year-old FFQ, and focused on who completed the TMD pain screener at age 18 and above	**Total sample*:*** 11.982 **Painful TMD** (n = 3163) 2449 (77.4%) women 714 (22.5%) men **No TMD** (n = 8819) 5297 (60.1%) women 3522 (39.9%) men	FFQ HEI	In unadjusted analysis, higher dietary quality at age 14 was associated with increased odds of painful TMD (OR = 1.14, CI: 1.01–1.29). After adjusting this association was not significant HEI and painful TMD: associations were not significant in adjusted models For dietary domains, no associations remained statistically significant after adjustment Pain frequency over the last 6 months: sugar was significantly associated with painful TMD in adjusted analysis (OR = 1.05, CI: 1.00–1.10)	4 years
Nogueira et al., 2025 [24]	Cross sectional	Women with main complain of myalgia or no history of TMD	DC/TMD	**G1 (TMD):** 31.9 ± 8.04 **G2 (control):** 33.5 ± 9.13	**Total sample:** 84 Only women **G1 (TMD)**: 68 **G2 (control):** 16	Three R24h food recalls (on non-consecutive days) and application of the (MSM) to estimate the usual intake	**G1**: Showed lower usual intake of cholesterol (*p* = 0.005), sodium (*p* = 0.014), omega-3 (*p* = 0.028), omega-6 (*p* = 0.002), folate (*p* = 0.002), magnesium (*p* = 0.029), selenium (*p* = 0.002) and higher usual intake of trans fat (*p* = 0.027) compared to G2. Also, **G1** showed higher prevalence of inadequacy of selenium (*p* = 0.010) and folate (*p* = 0.005) intake compared to G2 Dietary intake inadequacies in both groups: None of the participants presented adequate fiber intake. Vitamin D had a 100% prevalence of inadequacy in both groups, while vitamin E had 100% inadequacy for the asymptomatic group and 97% for the **G1** group. Almost all participants (95.6%) of the **G1** group had inadequate folate intake, and prevalence of inadequate magnesium intake was nearly 90%. Regarding omega 3, none of the participants in asymptomatic group had an adequate intake, while only 1.5% of the **G1** group achieved the reference value	No
Shimada et al., 2016 [22]	RCT/Cross-over	Myofascial pain	DC/TMD	Myofascial TMD: 29.0 ± 8.8 Healthy: 28.9 ± 10.7	**Total sample:** 24 **Women:** 20 (10 with myofascial TMD; and 10 healthy) **Men:** 4 (2 with myofascial TMD; and 2 healthy	Mixture in 400 ml sugar-free lemon soda: MSG (150 mg/kg) or sodium chloride 24 mg/kg (placebo) Food diary 24 h prior experiment	*Dialysate:* Concentration of glutamate was significantly higher in patients at 40 (p = 0.01) and 80 (p = 0.02) min after injection *Plasma*: Glutamate levels significantly increased 30 min after ingestion in the MSG session, in both groups and were significantly higher than the placebo *Saliva:* Glutamate levels significantly increased 30 min after ingestion in the MSG session, in both groups and were significantly higher than the placebo	No

*FFQ* food-frequency questionnaire, *DC/TMD* Diagnostic Criteria for Temporomandibular Disorders, *RDC/TMD* Research Diagnostic Criteria for Temporomandibular Disorders, *MSG* muscle glutamate concentration, *RCT* randomized controlled trials, *TMJ* temporomandibular joint, *MMO* maximal mouth opening, *VAS* visual analog scale, *SSI* Symptom Severity Index, *PLCS* Prospective and Longitudinal Cohort Study, *CS* Cross sectional, *MSM* Multiple Source Method, *HEI* Healthy Eating Index

The included populations varied considerably with respect to age, clinical characteristics, and study settings. One study investigated adolescents aged 12 to 17 years from the general population and examined the relationship between dietary habits and symptoms of temporomandibular disorders [21]. Another cross-sectional study included older adults aged 60 to 71 years and explored associations between dietary guideline compliance, oral health, and general health status [45]. Two large prospective cohort studies evaluated dietary exposures and their association with painful TMD later in life using data from the Danish National Birth Cohort [46, 48]. In addition, one observational study compared the dietary profile of women with chronic muscle TMD with that of asymptomatic individuals [49]. Two experimental studies were also included. One randomized controlled trial evaluated the effects of vitamin D supplementation compared with diclofenac sodium treatment in patients with myofascial TMD [47]. Another experimental crossover study assessed the effects of monosodium glutamate ingestion on muscle glutamate concentrations and pain sensitivity in individuals with myofascial TMD [22].

Diagnostic criteria for TMD differed between studies. Some studies used standardized diagnostic protocols such as the Diagnostic Criteria for Temporomandibular Disorders (DC/TMD) or validated TMD screening instruments, whereas others relied on clinical examinations combined with questionnaires assessing symptoms or functional limitations. Dietary exposures were assessed using several approaches, including food frequency questionnaires, dietary recall methods, dietary indices such as the Healthy Eating Index, and questionnaires specifically targeting dietary habits or food consumption patterns.

Due to heterogeneity in study designs, populations, and dietary assessments, a quantitative synthesis was not feasible. In addition, the studies evaluated substantially different dietary exposures and outcome domains, precluding meaningful quantitative pooling of results. Therefore, the findings are presented as a narrative synthesis.

### Narrative synthesis

Overall, the included studies indicate that the association between dietary factors and painful TMD remains complex and not yet fully clarified. The prospective cohort studies did not demonstrate that either prenatal or adolescent dietary quality independently predicted painful TMD after adjustment for confounding factors [46, 48].

In contrast, the cross-sectional studies suggested that specific dietary patterns, rather than overall dietary quality, may be related to selected TMD-related symptoms. In adolescents, frequent intake of hard food items was associated with joint clicking and temporomandibular joint pain, although no consistent association was observed between dietary habits and TMD symptoms overall [21]. In women with chronic muscle TMD, lower intake of several nutrients and a higher prevalence of nutrient inadequacies were observed compared with asymptomatic controls [49]. Moreover, in older adults, poorer compliance with dietary guidelines was associated with worse general health status, while orofacial pain was also independently associated with poorer health outcomes [45]. Although the latter study did not investigate painful TMD specifically, it suggests that diet and orofacial pain may interact within a broader health-related context. Furthermore, experimentally induced monosodium glutamate ingestion increased glutamate concentrations and spontaneous pain intensity in patients with myofascial TMD, suggesting that certain dietary compounds may modulate pain sensitivity in susceptible individuals [22].

The only randomized controlled trial did not show any significant between-group differences in subjective pain between vitamin D supplementation and diclofenac sodium treatment, although some functional improvement was observed in the vitamin D group [47].

Taken together, these findings suggest that while some dietary components or nutritional patterns may influence TMD-related symptoms or pain responses, the current evidence remains heterogeneous and does not allow firm conclusions regarding a clear dietary association or treatment approach for painful TMD.

## Discussion

The present systematic review investigated the potential relationship between dietary factors and painful TMD. Overall, the available evidence suggests that while certain dietary components or nutritional factors may be associated with specific TMD-related symptoms or pain responses, the current literature remains limited and heterogeneous. Prospective cohort studies did not demonstrate a consistent association between overall dietary quality and painful TMD, whereas cross-sectional and experimental studies indicated that specific dietary exposures or nutrient profiles may influence symptom patterns or pain sensitivity in some patient groups. These findings, coming from studies with low risk of bias, highlight the complexity of the relationship between diet and painful TMD and suggest that dietary factors may represent one component within the broader biopsychosocial framework of TMD. Importantly, the included studies addressed two distinct research questions. Some studies investigated whether dietary factors or nutritional status were associated with painful TMD or TMD-related symptoms, whereas others explored how painful TMD may influence dietary behaviors, food choices, and nutritional intake. These represent different directions of association and should be interpreted separately.

Diet- and nutrition-related mechanisms in painful TMD appear biologically plausible, although current evidence remains limited and heterogeneous [21, 22, 46, 48, 49]. Mechanical factors may partly explain this relationship. Prolonged mastication of harder foods increases masticatory muscle activity and joint loading, which may exacerbate pain in susceptible individuals or in the presence of ongoing painful TMD. Nevertheless, epidemiological studies have generally reported small and inconsistent associations between specific food textures and TMD pain [21]. Beyond mechanical and neurochemical mechanisms nutritional factors may also influence pain modulation pathways. Experimental evidence also suggests that ingestion of monosodium glutamate can increase interstitial glutamate concentrations in the masseter and transiently intensify ongoing myofascial TMD pain, supporting a possible role for peripheral excitatory signaling in symptom modulation [22]. Furthermore, women with chronic myogenous TMD have been reported to present lower intake of nutrients involved in neuromodulation and oxidative stress regulation, alongside with higher intake of trans-fatty acids, suggesting a potential link between dietary composition and altered pain processing [49]. Similarly, a cross-sectional study conducted in Mediterranean populations reported that individuals with painful TMD consumed less healthy food patterns, which emerged as one of the strongest predictors associated with painful TMD, although the study presented some methodological limitations [23]. However, prospective investigations evaluating overall diet quality indicate at most weak and inconsistent associations with later painful TMD, suggesting that any dietary contribution is likely modest and embedded within a broader multifactorial biopsychosocial context [46, 48].

In line with this, the concept of an “inflammatory diet” in painful TMD can currently only be inferred indirectly and has not been directly demonstrated as an independent etiologic factor [46, 48, 49]. Nutritional inadequacies observed in patients with chronic myogenous TMD, such as lower intake of omega-3 and omega-6 fatty acids, folate, magnesium, and selenium combined with higher trans-fat consumption, may be compatible with a more pro-inflammatory nutritional profile; however, these cross-sectional observations do not clarify whether such dietary patterns contribute to pain mechanisms or instead arise as a consequence of pain-related behavioral adaptations [49]. More broadly, nutrition has been proposed as a modulator of chronic pain through several biological pathways, including regulation of inflammatory mediators, oxidative stress, and neuromodulatory processes [50–52]. These results aligned with a cross-sectional study exhibiting a tendency of painful TMD patients toward greater intake of proinflammatory dietary compared with controls [23]. Diets characterized by high intake of refined sugars or saturated fats may promote systemic inflammatory responses through increased production of cytokines such as TNF-α and interleukin-6, which have been implicated in nociceptive sensitization [53, 54]. Experimental models also suggest that high-fat diets may increase pain sensitivity, possibly through metabolic and neuroimmune mechanisms [55]. Conversely, several micronutrients, including B-complex vitamins, magnesium, and vitamin D, have been associated with neuromodulatory and anti-inflammatory effects that may influence pain perception, although clinical evidence remains inconsistent [14, 25, 56]. Large prospective cohort studies evaluating overall diet quality during adolescence and pregnancy did not identify robust associations with later painful TMD, although some dietary components potentially related to pro- or anti-inflammatory exposure showed weak and inconsistent associations with headache outcomes [46, 48]. Taken together, these findings suggest that inflammation-related dietary components may be relevant to symptom expression or pain-related mechanisms in susceptible individuals, but the evidence available in this review remains limited and heterogeneous, and does not support an “inflammatory diet” as an independent risk factor for painful TMD [46, 48, 49].

Further caution is warranted when interpreting these findings because the limited number of available studies address distinct nutritional dimensions and rely on heterogeneous methodological designs, precluding firm inferences regarding pain modulation in TMD [22, 49]. For example, the experimental study involving monosodium glutamate evaluated the acute effects to a single excitatory amino acid under controlled conditions, whereas the dietary profile studies assessed habitual intake of multiple nutrients with indirect relevance to pain-related mechanisms. Importantly, none of these investigations were designed to test targeted nutritional interventions or to evaluate changes in clinical outcomes over time [22, 49]. Therefore, although these findings support the biological plausibility that specific dietary components or nutrient inadequacies may influence pain perception or symptom burden, they remain insufficient to establish a specific mechanistic model or to justify dietary strategies as established approaches to pain management in painful TMD.

Another relevant aspect concerns the possibility that dietary pattern observed in painful TMD may partially reflect behavioral adaptation to mastication-related pain rather than only underlying nutritional exposure [21, 49]. Cross-sectional evidence indicates that patients with TMD frequently report pain during chewing and greater jaw functional limitation, which may lead them to modify food texture by preferring softer food, cutting food into smaller pieces, and avoiding harder foods that may exacerbate symptoms [17, 20, 21, 49]. Such pain-driven adaptations may reduce the intake of fibrous foods that require greater chewing effort, including fruits, vegetables and foods rich in dietary fiber, potentially affecting overall dietary quality [7]. Consequently, observed differences in nutritional intake may represent both potential contributors to symptom modulation and consequences of pain-related functional avoidance. At present, however, the available evidence does not allow these directions of association to be clearly disentangled [21, 49].

### Methodological considerations

Several methodological considerations should be considered when interpreting the findings of this review. A strength of the present study is the systematic search strategy across multiple databases and the predefined inclusion criteria, as well as the use of a standardized tool to assess risk of bias. Only studies judged to have a low risk of bias were included in the final synthesis, which strengthens the reliability of the findings. However, several limitations should also be acknowledged. The included studies differed substantially in terms of study design, populations, diagnostic criteria for TMD, and methods used to assess dietary exposures, which limited comparability between studies and precluded quantitative synthesis. Furthermore, the included studies evaluated heterogeneous outcome domains, including TMD-related symptoms, pain intensity, nutritional intake, dietary quality, and experimentally induced pain responses, further limiting direct comparisons across studies. In addition, just one study reported a sample size calculation, which probably makes most of the included studies unpowered. Furthermore, most studies were observational in nature, making it difficult to establish causal relationships between dietary factors and painful TMD. Dietary assessments were also based primarily on self-reported questionnaires, which may introduce recall bias or measurement errors. Consequently, the findings of this review should be interpreted with caution.

### Clinical implications and future research

From a clinical perspective, the current evidence suggests that dietary habits and nutritional status may be associated with painful TMD. However, the predominantly observational nature of the included studies precludes conclusions regarding causality or the role of dietary factors in the development of TMD. Clinicians should therefore remain aware that dietary behaviors and nutritional status should be regarded as potentially relevant clinical considerations rather than established therapeutics targets. Nevertheless, due to the limited and heterogeneous evidence, dietary recommendations for TMD management cannot currently be based on strong scientific evidence. Considering our findings, and in accordance with the INfORM/IADR key points for good clinical practice based on standard of care [57], patient-centered decision-making remains essential in the management of TMDs. In this context, diagnosis should be based on standardized and validated history taking and clinical assessment, which may include consideration of dietary habits and nutritional status when relevant to the patient´s clinical presentation.

Future research should aim to clarify the potential role of dietary factors in painful TMD through well-designed longitudinal and interventional studies. In particular, studies using standardized diagnostic criteria for TMD and validated dietary assessment methods would improve comparability across investigations. Furthermore, randomized controlled trials examining dietary modifications or nutritional interventions may help determine whether specific dietary patterns or nutrient intake can influence pain intensity, functional outcomes, or disease progression in patients with TMD.

## Conclusion

In conclusion, current evidence does not support a clear association between overall dietary quality and painful TMD. Although some studies suggest that specific dietary components or nutritional factors may influence TMD-related symptoms, these findings remain inconsistent and largely derived from heterogenous observational data. Therefore, the role of diet and nutrition in the development or modulation of painful TMD remains uncertain. Well-designed longitudinal and interventional studies are needed to clarify whether dietary factors contribute meaningfully to painful TMD pathophysiology or clinical.

## Supplementary Information

Supplementary Material 1.

Supplementary Material 2.

Supplementary Material 3.

## Data Availability

The datasets used and/or analysed during the current study are available from the corresponding author on reasonable request.
