# Supplementary material for: Can poor nutrition and diet influence temporomandibular disorder? A systematic review
Source: BMC Oral Health. 2026 Jun 23;26:1110. doi: 10.1186/s12903-026-08975-3 (PMC13289324; doi:10.1186/s12903-026-08975-3)
Supplement: Supplementary file 2 — Supplementary Material 2. [file 12903_2026_8975_MOESM2_ESM.docx]

**Supplemental table 1:** This file contains the main search strategies by the University Library search consulting group (AnnaMia Eborn Martinovic and Lovisa Liljegren). The literature search was performed in the following databases: Medline, Embase, Web of Science, Cinahl and Cochrane. The search was conducted 2024-04-12. After the original search was performed (12^th^ of April 2024) the search was last updated on 14^th^ of November 2025 by rerunning the searches and deduplicating against the previous results.

The search strategy was developed in Medline (Ovid) in collaboration with librarians at the Karolinska Institutet University Library. For each search concept Medical Subject Headings (MeSH-terms) and free text terms were identified. The search was then translated into the other databases. In total there were 7,760 unique hits.

1. Medline

| Interface: Ovid MEDLINE(R) ALL  Date of Search: 14 Nov 2025  Number of hits: 2,338  Comment: In Ovid, two or more words are automatically searched as phrases; i.e. no quotation marks are needed | Field labels   - exp/ = exploded MeSH term - / = non exploded MeSH term - .ti,ab,kf. = title, abstract and author keywords - adjx = within x words, regardless of order - * = truncation of word for alternate endings |
| --- | --- |
| \| 1 \| exp Craniomandibular Disorders/ \| 20,831 \| \| --- \| --- \| --- \| \| 2 \| exp Myofascial Pain Syndromes/ \| 7,070 \| \| 3 \| (Craniomandibular adj3 (arthropath* or disease* or disorder* or dysfuncti* or pain* or syndrom*)).ti,ab,kf. \| 722 \| \| 4 \| ((jaw or temporomandibular or tmj) adj3 (arthropath* or disease* or dislocation* or disorder* or dysfuncti* or luxation* or pain* or subluxation* or syndrom*)).ti,ab,kf. \| 16,961 \| \| 5 \| ((myofascial or orofacial) adj2 (pain* or syndrom* or trigger point*)).ti,ab,kf. \| 7,774 \| \| 6 \| (costen* syndrom* or tmd*).ti,ab,kf. \| 15,304 \| \| 7 \| Arthralgia/ \| 10,655 \| \| 8 \| Myalgia/ \| 3,138 \| \| 9 \| (arthralgia* or myalgia* or polyarthralgia*).ti,ab,kf. \| 22,936 \| \| 10 \| joint pain*.ti,ab,kf. \| 11,954 \| \| 11 \| (muscle adj2 (pain or soreness* or tenderness)).ti,ab,kf. \| 11,240 \| \| 12 \| or/1-11 \| 91,111 \| \| 13 \| exp Diet/ \| 360,769 \| \| 14 \| exp Food/ \| 1,597,796 \| \| 15 \| exp Feeding Behavior/ \| 207,349 \| \| 16 \| exp Eating/ \| 83,662 \| \| 17 \| exp Nutrients/ \| 639,511 \| \| 18 \| exp "Feeding and Eating disorders"/ \| 39,874 \| \| 19 \| (diet* or nutrition* or food*).ti,ab,kf. \| 1,629,540 \| \| 20 \| ((eating or feeding or alimentary) adj2 (behavio?r or pattern* or program* or habit*)).ti,ab,kf. \| 46,393 \| \| 21 \| (nutrient* or macronutrient* or micronutrient*).ti,ab,kf. \| 256,434 \| \| 22 \| (eating disorder* or feeding disorder* or anorexi* or bulimi* or binge eating or fasting).ti,ab,kf. \| 213,290 \| \| 23 \| or/13-22 \| 3,197,591 \| \| 24 \| exp Pain/ \| 495,477 \| \| 25 \| exp Inflammation/ \| 464,912 \| \| 26 \| (pain* or inflammat*).ti,ab,kf. \| 2,361,374 \| \| 27 \| or/24-26 \| 2,724,809 \| \| 28 \| 12 and 23 and 27 \| 3,329 \| \| 29 \| limit 28 to humans \| 2,731 \| \| 30 \| limit 29 to (case reports or editorial or letter) \| 393 \| \| 31 \| 29 not 30 \| 2,338 \| | |

2. Embase

| Interface: embase.com  Date of Search: 14 Nov 2025  Number of hits: 4,445  Comment: Emtree is the controlled vocabulary in Embase | Field labels   - /exp = exploded Emtree term - /de = non exploded Emtree term - ti,ab,kw = title, abstract and author keywords - NEAR/x = within x words, regardless of order - * = truncation of word for alternate endings |
| --- | --- |
| \| \| #1 \| 'temporomandibular joint disorder'/exp/mj \| 13,614 \| \| --- \| --- \| --- \| \| #2 \| 'myofascial pain'/exp \| 10,073 \| \| #3 \| (craniomandibular NEAR/3 (arthropath* OR disease* OR disorder* OR dysfuncti* OR pain* OR syndrom*)):ti,ab,kw \| 847 \| \| #4 \| ((jaw OR temporomandibular OR tmj) NEAR/3 (arthropath* OR disease* OR dislocation* OR disorder* OR dysfuncti* OR luxation* OR pain* OR subluxation* OR syndrom*)):ti,ab,kw \| 20,164 \| \| #5 \| ((myofascial OR orofacial) NEAR/2 (pain* OR syndrom* OR 'trigger point*')):ti,ab,kw \| 10,959 \| \| #6 \| 'costen* syndrom*':ti,ab,kw OR tmd*:ti,ab,kw \| 18,244 \| \| #7 \| 'arthralgia'/de \| 93,284 \| \| #8 \| 'myalgia'/de \| 82,589 \| \| #9 \| arthralgia*:ti,ab,kw OR myalgia*:ti,ab,kw OR polyarthralgia*:ti,ab,kw \| 42,912 \| \| #10 \| 'joint pain*':ti,ab,kw \| 21,710 \| \| #11 \| (muscle NEAR/2 (pain OR soreness* OR tenderness)):ti,ab,kw \| 17,136 \| \| #12 \| #1 OR #2 OR #3 OR #4 OR #5 OR #6 OR #7 OR #8 OR #9 OR #10 OR #11 \| 220,985 \| \| #13 \| 'diet'/exp/mj \| 169,105 \| \| #14 \| 'food'/exp/mj \| 712,763 \| \| #15 \| 'feeding behavior'/exp/mj \| 85,945 \| \| #16 \| 'eating'/exp/mj \| 12,235 \| \| #17 \| 'nutrient'/exp/mj \| 16,367 \| \| #18 \| 'eating disorder'/exp/mj \| 48,409 \| \| #19 \| diet*:ti,ab,kw OR nutrition*:ti,ab,kw OR food*:ti,ab,kw \| 2,079,612 \| \| #20 \| ((eating OR feeding OR alimentary) NEAR/2 (behavio$r OR program* OR pattern* OR habit*)):ti,ab,kw \| 61,898 \| \| #21 \| nutrient*:ti,ab,kw OR macronutrient*:ti,ab,kw OR micronutrient*:ti,ab,kw \| 302,076 \| \| #22 \| 'eating disorder' OR 'feeding disorder' OR anorexi* OR bulimi* OR 'binge eating' or fasting:ti,ab,kw \| 406,596 \| \| #23 \| #13 OR #14 OR #15 OR #16 OR #17 OR #18 OR #19 OR #20 OR #21 OR #22 \| 3,056,382 \| \| #24 \| 'pain'/exp/mj \| 595,520 \| \| #25 \| 'inflammation'/exp/mj \| 3,519,817 \| \| #26 \| pain*:ti,ab,kw OR inflammat*:ti,ab,kw \| 3,521,859 \| \| #27 \| #24 OR #25 OR #26 \| 6,274,405 \| \| #28 \| #12 AND #23 AND #27 \| 7,999 \| \| #29 \| #28 AND ('case report'/de OR 'nonhuman'/de) \| 2,485 \| \| #30 \| #28 NOT #29 \| 5,514 \| \| #31 \| #30 AND ('conference abstract'/it OR 'conference paper'/it OR 'editorial'/it OR 'letter'/it OR 'note'/it OR 'tombstone'/it) \| 1,069 \| \| #32 \| #30 NOT #31 \| 4,445 \| \| \| --- \| --- \| --- \| --- \| --- \| --- \| --- \| --- \| --- \| --- \| --- \| --- \| --- \| --- \| --- \| --- \| --- \| --- \| --- \| --- \| --- \| --- \| --- \| --- \| --- \| --- \| --- \| --- \| --- \| --- \| --- \| --- \| --- \| --- \| --- \| --- \| --- \| --- \| --- \| --- \| --- \| --- \| --- \| --- \| --- \| --- \| --- \| --- \| --- \| --- \| --- \| --- \| --- \| --- \| --- \| --- \| --- \| --- \| --- \| --- \| --- \| --- \| --- \| --- \| --- \| --- \| --- \| --- \| --- \| --- \| --- \| --- \| --- \| --- \| --- \| --- \| --- \| --- \| --- \| --- \| --- \| --- \| --- \| --- \| --- \| --- \| --- \| --- \| --- \| --- \| --- \| --- \| --- \| --- \| --- \| --- \| --- \| | |

3. Cochrane Library

| Interface: Wiley  Date of Search: 14 Nov 2025  Number of hits: 1,862 | Field labels   - ti,ab,kw = title, abstract and author keywords - NEAR/x = within x words, regardless of order - * = truncation of word for alternate endings |
| --- | --- |
| \| #1 \| [mh "Craniomandibular Disorders"] \| 1,325 \| \| --- \| --- \| --- \| \| #2 \| [mh "Myofascial Pain Syndromes"] \| 1,809 \| \| #3 \| ((Craniomandibular NEAR/3 (arthropath* OR disease* OR disorder* OR dysfuncti* OR pain* OR syndrom*))):ti,ab,kw \| 81 \| \| #4 \| ((jaw or temporomandibular or tmj) NEAR/3 (arthropath* or disease* or dislocation* or disorder* or dysfuncti* or luxation* or pain* or subluxation* or syndrom*)):ti,ab,kw \| 3,022 \| \| #5 \| ((myofascial or orofacial) NEAR/2 (pain* or syndrom* or trigger point*)):ti,ab,kw \| 3,493 \| \| #6 \| ((costen* NEXT syndrom*) OR tmd):ti,ab,kw \| 1,441 \| \| #7 \| [mh Arthralgia] \| 2,901 \| \| #8 \| [mh Myalgia] \| 944 \| \| #9 \| (arthralgia* or myalgia* or polyarthralgia*):ti,ab,kw \| 11,079 \| \| #10 \| (joint NEXT pain*):ti,ab,kw \| 3,241 \| \| #11 \| (muscle NEAR/2 (pain or soreness* or tenderness)):ti,ab,kw \| 6,303 \| \| #12 \| #1 OR #2 OR #3 OR #4 OR #5 OR #6 OR #7 OR #8 OR #9 OR #10 OR #11 \| 24,818 \| \| #13 \| [mh Diet] \| 26,389 \| \| #14 \| [mh Food] \| 48,779 \| \| #15 \| [mh “Feeding Behavior”] \| 13,146 \| \| #16 \| [mh “Eating”] \| 5,026 \| \| #17 \| [mh Nutrients] \| 6,495 \| \| #18 \| [mh “Feeding and Eating Disorders”] \| 2,677 \| \| #19 \| (diet* or nutrition* or food*):ti,ab,kw \| 191,757 \| \| #20 \| ((eating or feeding or alimentary) NEAR/2 (behavio?r or program* or habit* or pattern*)):ti,ab,kw \| 10,609 \| \| #21 \| (nutrient* or macronutrient* or micronutrient*):ti,ab,kw \| 15,835 \| \| #22 \| ((eating or feeding) NEXT disorder*):ti,ab,kw \| 4,291 \| \| #23 \| (anorexi* or bulimi* or “binge eating” or fasting):ti,ab,kw \| 56,552 \| \| #24 \| #13 OR #14 OR #15 OR #16 OR #17 OR #18 OR #19 OR #20 OR #21 OR #22 OR #23 \| 240,384 \| \| #25 \| [mh Pain] \| 74,840 \| \| #26 \| [mh Inflammation] \| 16,869 \| \| #27 \| (pain* or inflammat*):ti,ab,kw \| 383,477 \| \| #28 \| #25 OR #26 OR #27 \| 397,336 \| \| #29 \| #12 AND #24 AND #28 \| 1,862 \| | |

4. Web of Science Core Collection

| Interface: Clarivate Analytics  Editions = A&HCI , ESCI , SCI-EXPANDED , SSCI  Date of Search: 14 Nov 2025  Number of hits: 1,965 | Field labels   - TS/Topic = title, abstract, author keywords and Keywords Plus - NEAR/x = within x words, regardless of order - * = truncation of word for alternate endings   Note: the *Exact search*-function was used for all the searches |
| --- | --- |
| \| 1 \| TS=((craniomandibular NEAR/2 (arthropath* OR disease* OR disorder* OR dysfuncti* OR pain* OR syndrom*)) \| 989 \| \| --- \| --- \| --- \| \| 2 \| TS=((jaw OR temporomandibular OR tmj) NEAR/2 (arthropath* OR disease* OR dislocation* OR disorder* OR dysfuncti* OR luxation* OR pain* OR subluxation* OR syndrom*)) \| 16,665 \| \| 3 \| TS=((myofascial OR orofacial) NEAR/1 (pain* OR syndrom* OR "trigger point*")) \| 10,121 \| \| 4 \| TS=(("costen* syndrom*" OR tmd*)) \| 27,711 \| \| 5 \| TS=((arthralgia* OR myalgia* OR polyarthralgia*)) \| 21,837 \| \| 6 \| TS=("joint pain*") \| 12,368 \| \| 7 \| TS=((muscle NEAR/1 (pain OR soreness* OR tenderness))) \| 12,887 \| \| 8 \| #7 OR #6 OR #5 OR #4 OR #3 OR #2 OR #1 \| 87,924 \| \| 9 \| TS=((diet* OR nutrition* OR food*)) \| 2,773,536 \| \| 10 \| TS=((eating OR feeding OR alimentary) NEAR/1 (behavio$r OR program* OR habit* OR pattern*)) \| 85,918 \| \| 11 \| TS=((nutrient* OR macronutrient* OR micronutrient*)) \| 566,178 \| \| 12 \| TS=("eating disorder*" or "feeding disorder*" or anorexi* or bulimi* or “binge eating” or fasting)) \| 243,743 \| \| 13 \| #9 OR #10 OR #11 OR #12 \| 3,332,822 \| \| 14 \| TS=(pain* OR inflammat*) \| 2,819,171 \| \| 15 \| #13 AND #14 AND #8 \| 1,965 \| | |

5. Cinahl

| Interface: Ebsco  Date of Search: 14 Nov 2025  Number of hits: 1,150 | Field labels   - MH+ = exploded Cinahl Heading - MH = non exploded Cinahl Heading - XB = title/abstract - Nx = within x words, regardless of order - * = truncation of word for alternate endings   Note: sometimes “quotation marks” are needed for single search terms to avoid automatic term mapping (lemmatization)1 |
| --- | --- |
| \| S1 \| (MH "Craniomandibular Disorders+") \| 6,238 \| \| --- \| --- \| --- \| \| S2 \| (MH "Myofascial Pain Syndromes+") \| 3,669 \| \| S3 \| (MH "Temporomandibular Joint Syndrome") \| 658 \| \| S4 \| XB (craniomandibular N2 (arthropath* or disease* or disorder* or dysfuncti* or pain* or syndrom*)) \| 83 \| \| S5 \| XB ((jaw or temporomandibular or tmj) N2 (arthropath* or disease* or dislocation* or disorder* or dysfuncti* or luxation* or pain* or subluxation* or syndrom*)) \| 5,460 \| \| S6 \| XB ((myofascial or orofacial) N1 (pain* or syndrom* or trigger point*)) \| 3,664 \| \| S7 \| XB ("costen* syndrom*" or tmd*) \| 3,110 \| \| S8 \| (MH "Arthralgia") \| 4,100 \| \| S9 \| (MH "Muscle Pain") \| 3,867 \| \| S10 \| XB (arthralgia* or myalgia* or polyarthralgia*) \| 4,503 \| \| S11 \| XB ("joint pain*") \| 3,630 \| \| S12 \| XB ( muscle N1(pain or soreness* or tenderness)) \| 4,223 \| \| S13 \| S1 OR S2 OR S3 OR S4 OR S5 OR S6 OR S7 OR S8 OR S9 OR S10 OR S11 OR S12 \| 27,790 \| \| S14 \| (MH "Diet+") \| 153,179 \| \| S15 \| (MH "Food+") \| 211,701 \| \| S16 \| (MH "Eating Behavior+") \| 57,400 \| \| S17 \| (MH "Eating") \| 7,757 \| \| S18 \| (MH "Nutrients+") \| 13,067 \| \| S19 \| (MH "Eating Disorders+") \| 23,969 \| \| S20 \| XB (diet* or nutrition* or food*) \| 350,264 \| \| S21 \| XB ((eating or feeding or alimentary) N1 (behavio#r or program* or pattern* or habit* )) \| 12,547 \| \| S22 \| XB (nutrient* or macronutrient* or micronutrient*) \| 33,931 \| \| S23 \| XB ("eating disorder*" or "feeding disorder*" or anorexi* or bulimi* or “binge eating” or fasting) \| 56,051 \| \| S24 \| S14 OR S15 OR S16 OR S17 OR S18 OR S19 OR S20 OR S21 OR S22 OR S23 \| 562,912 \| \| S25 \| (MH "Pain+") \| 248,721 \| \| S26 \| (MH "Inflammation+") \| 84,915 \| \| S27 \| XB (pain* or inflammat*) \| 505,807 \| \| S28 \| S25 OR S26 OR S27 \| 626,285 \| \| S29 \| S13 AND S24 AND S28 \| 1,150 \| | |
